# Networks in the Field of Tourette Syndrome

**DOI:** 10.3389/fneur.2021.624858

**Published:** 2021-04-13

**Authors:** Alexander Kleimaker, Maximilian Kleimaker, Amelie Behm, Anne Weissbach, Tobias Bäumer, Christian Beste, Veit Roessner, Alexander Münchau

**Affiliations:** ^1^Institute of Systems Motor Science, University of Lübeck, Lübeck, Germany; ^2^Department of Neurology, University Hospital Schleswig-Holstein, Lübeck, Germany; ^3^Institute of Neurogenetics, University of Lübeck, Lübeck, Germany; ^4^Department of Child and Adolescent Psychiatry, Faculty of Medicine, Technische Universität (TU) Dresden, Dresden, Germany

**Keywords:** Gilles de la Tourette syndrome, European Multicenter Tics in Children Studies, European Society for The Study of Tourette Syndrome, the Tourette Association of America, research networks

## Abstract

Gilles de la Tourette syndrome (TS) is a neuropsychiatric neurodevelopmental disorder with the cardinal clinical features of motor and phonic tics. Clinical phenomenology can be complex since, besides tics, there are other features including premonitory urges preceding tics, pali-, echo-, and coprophenomena, hypersensitivity to external stimuli, and symptom dependency on stress, attention, and other less well-defined factors. Also, the rate of comorbidities, particularly attention deficit hyperactivity disorder and obsessive-compulsive disorder, is high. Mirroring the complexities of the clinical course and phenomenology, pathophysiological findings are very diverse, and etiology is disputed. It has become clear, though, that abnormalities in the basal ganglia and their connections with cortical areas are key for the understanding of the pathophysiology and as regards etiology, genetic factors are crucial. Against this background, both adequate clinical management of TS and TS-related research require multidisciplinary preferably international cooperation in larger groups or networks to address the multiple facets of this disorder and yield valid and useful data. In particular, large numbers of patients are needed for brain imaging and genetic studies. To meet these requirements, a number of networks and groups in the field of TS have developed over the years creating an efficient, lively, and supportive international research community. In this review, we will provide an overview of these groups and networks.

## TS As A Prototype Neuropsychiatric Neurodevelopmental Spectrum Disorder

Gilles de la Tourette syndrome (TS) is a multifaceted neuropsychiatric disease defined by multiple motor and at least one phonic tic starting before the age of 18 and lasting for at least 1 year (1). Disease onset is usually in early childhood (2). Clinical phenomenology varies widely with tic repertoire reaching from simple motor and phonic tics including, for instance, eye blinking, mouth pouting, throat clearing, or sniffing to complex movements or vocalizations like body turning or squatting, or the utterance of single words or phrases (3). Although public perception is strongly dominated by coprophenomena, i.e., the utterance of swear words (coprolalia) or the execution of obscene gestures (copropraxia), coprophenomena are present in only about 20% of patients (4, 5).

While first motor tics usually occur around the age of 6, phonic tics tend to emerge several years later (6). However, there is also a group of children who first develop phonic tics, which may or may not be followed by the occurrence of motor tics. Both tic repertoire and intensity fluctuate over time, i.e., they “wax and wane” (6). In the majority of cases, tics are preceded by various perceptual phenomena referred to as premonitory urges (7). They typically decrease after tic execution (6, 8–10). Moreover, tic severity is influenced by cognitive processes. For instance, while there is an ongoing discussion on what kind of stress might result in an increase or decrease of tic severity (11), it is undisputed that distraction can lead to an amelioration of symptoms (12, 13). Many TS patients suffer from psychiatric comorbidities. About 90% of TS patients have comorbidities including attention deficit, hyperactivity disorder, and obsessive-compulsive disorder (40%) (14).

In most cases, the disease course is benign. Following a peak in pre-puberty, or puberty, symptoms usually considerably improve until the age of 18 (6). Thus, more often than not, therapy concepts based on counseling are sufficient. However, TS can impair psychosocial development, can lead to significant secondary morbidity and impair quality of life (15, 16). In about 20% of cases symptoms persist into adulthood and then often affect personal life markedly (15, 17, 18). However, it has to be pointed out that data on the clinical course, particularly evolution of symptoms, severity fluctuations and percentage of remissions are limited, particularly because longitudinal studies are scarce.

The gold standard clinical intervention is comprehensive behavioral intervention for tics including habit reversal therapy with the core components awareness training and the acquisition of a competing response that is incompatible with the tic (19). Exposure and response prevention is an alternative behavioral intervention (20). The mainstay of pharmacological treatment are antipsychotic drugs including tiapride or aripiprazole (21, 22). When tics affect few muscles or muscle groups, botulinum toxin injections might be used (23, 24). Deep brain stimulation is a treatment option in severely affected TS patients refractory to conventional therapy. Most frequently used targets are the centro-median-parafascicular complex of the intralaminar thalamus (25) and the globus pallidus internus (26–28).

Regarding the underlying pathophysiology, there is still no uniform concept and several explanatory approaches coexist. Research work dates back to the late nineteenth century. While in the early part of the twentieth century until the 1970s, TS was primarily considered a psychiatric disorder (29), neurophysiological findings (30), as well as successful medical treatment with antipsychotic drugs (22) and accumulating genetic data (31), have changed this view (32). TS is now conceptualized as a neurodevelopmental neuropsychiatric spectrum disorder predominantly of genetic origin (33). The role of environmental factors and their interaction with genetic predisposition, though, is unclear.

Given its nature as a multifaceted, often complex disorder affecting both children and adults, it comes with no surprise that abnormalities in patients with TS have been delineated in different research fields. For instance, at a neuroanatomical level, alterations have been described in cortical regions including reduced gray matter volume in prefrontal and sensorimotor areas (34), or the basal ganglia (e.g., reduced caudate nucleus volume) (34, 35).

Neurophysiologically, sensorimotor abnormalities, including altered sensory processing such as deficient prepulse inhibition (36) serving as a measure for sensorimotor gating (37), and altered short afferent inhibition as a measure for sensorimotor integration (38, 39), are a common theme in TS research.

In the field of cognitive psychology, a higher tendency for habit formation (40), abnormalities concerning interoceptive awareness (41), and altered inhibitory control (42) have emerged as relevant findings in TS. Also, abnormally increased binding between actions and perceptions has been shown in these patients (43). Furthermore, many family studies have been conducted suggesting that TS is largely a genetic disorder (44).

Against the background of the natural course of TS and numerous findings derived from various research fields, it has become clear that different specialties, particularly neurology, child and adolescent and adult psychiatry, pediatric neurology, (neuro-) genetics, and research teams covering different fields of neuroscience, e.g., neuroanatomy, neuroimaging, neurophysiology, neurogenetics, (cognitive) psychology, need to join forces to better understand the neurobiological background of TS over the lifespan and to develop more individualized management strategies. Mutual exchange of information between specialties and research fields has become a prerequisite for innovative and efficient research.

## Need for Interdisciplinary and Globally Connected Networks

For a long time, scientific findings and breakthroughs have been associated with exceptional scientifically outstanding individuals. Gilles de la Tourette, who first delineated TS as a neuropsychiatric syndrome, is one of many examples (45). However, as scientific knowledge and therapeutic options are constantly accumulating globally rather than locally, there is a strong need for structures and organizations connecting the different researchers to facilitate international collaboration and exchange.

This is corroborated by the fact that the number of multiauthored publications has increased (46). Before the Second World War, most cited papers were written by single authors. Since the 1950s, the number of collaborative papers has risen steadily (47). Of note, research dealing with scientific and economic networks and collaborations has also evolved with focuses not only on the number of people engaged within networks but also their composition and inner structure (48).

Two opposing views have emerged (48). On the one hand, high diversity of network participants is considered advantageous, potentially increasing the spectrum of skills and perspectives and thus fostering the capacity for problem solving (49). Also, it is argued that high diversity within a team results in cross-fertilization processes due to a combination of different perspectives and expertise (50–52). On the other hand, it has been put forward that communication between team members in more heterogeneous groups might be more complicated and efficiency reduced due to a lack of shared identity compared to homogeneous groups (48).

In addition to these more general and theoretical considerations, optimal group, i.e., network composition, is obviously also determined by the area of research and the overarching aims of respective networks. Given the multifaceted nature of TS with respect to clinical manifestation including a large age range and etiological/pathophysiological background, TS research networks should ideally be composed of international, multiprofessional groups.

## Networks in the Field of Tourette Syndrome

### Patient Organizations

First and foremost, a number of national patient organization promote and support patient-related matters and also international research. For a comprehensive overview of various activities in many different countries, please see https://www.essts.org/directory.

Examples are the Tourette Association of America (USA), the Caribbean Tourette Association, Asociación Argentina para el Síndrome de Tourette, the Israel Tourette Association, the Tic Disorders and Tourette Syndrome Association of China, the Tourette Association Japan, the Tourette Syndrome Association of Australia, Tourette Action (UK), the Association francaise du Syndrome de Gilles de la Tourette, and the German Tourette Association.

For instance, founded in 1972, The Tourette Association of America (TAA) is the largest TS patient organization worldwide (https://philanthropynewsdigest.org/npo-spotlight/tourette-association-of-america). It serves the purpose of financing research, educating both patients and professionals, and creating awareness of TS. Up to now, more than $22 million have been awarded to over 450 research projects in 16 countries (https://tourette.org/about-us/mission-and-history/). In addition, public relations activities are a major component of its work. In this context, the Tourette Association of America Youth Ambassador Program was created. In this program, children suffering from TS are being trained in proliferating up-to-date information on their disease in their social surroundings (https://tourette.org/about-tourette/overview/living-tourette-syndrome/teens-13-17/youth-ambassador-program/).

In the UK, in 1980, the Tourettes Action was brought into being by a group of parents of affected infants, first known as Tourette Syndrome Association (UK). The primary objective of this organization was to provide mutual assistance for coping with everyday life and to promote social acceptance of TS patients. In 2006, the association moved its headquarters to London, and in 2008, the name was changed in Tourettes Action. Besides supporting patients and relatives, the aim has also become to promote research. Tourette Action is also active in organizing workshops, conferences, and activities for young people and offers subsided CBITS training for clinicians and professionals across the UK (Tourettes-action.org.uk).

The German Tourette Association (Tourette-gesellschaft.de) was founded in 1993. Its declared aim is to provide current and valid information on TS and to communicate treatment options. The association has, for instance, developed a geographical map providing an overview of specialists and clinicians, simplifying the search for medical support. In addition, it provides help in finding support groups and organizes activities for young people with TS.

### Outreach Activities

An example of TS-related outreach activities by professional artists is Manhattan's La MaMa Experimental Theater Club that presented the play *The Elephant in Every Room I Enter*, a play about the challenges of working as an actor with Tourette Syndrome. The Agency of Surplus, a neuroscience/theater/performance/film group based in Hamburg, Germany, can serve as an example of a multiprofessional network comprising professionals from the field of neurology, neuroscience, philosophy, theater sciences/performance studies, stage design, and film aiming at promoting and proliferating outreach activities related to TS. The Agency of Surplus has produced theater plays where patients with TS participated as performers. For instance, the theater play “Theater of disgraceful people” (*Das Theater der infamen Menschen*) (https://www.ballhausost.de/produktionen/theater-of-disgraceful-people/) was part of the official program of the International Parkinson and Movement Disorder Society Congress in Berlin in 2016. In 2020, the Agency of Surplus produced an international documentary film (“TICS”), a road movie, where three patients with TS first visited the Salpêtrière Hospital in Paris, where Tourette syndrome was first described and which is still a major center for Tourette research worldwide, then traveled to the Universities of Cologne, Hannover, and Lübeck (Germany) and ultimately to Lapland, where the reception of tics in a different social context was explored. The documentary also provides information on behavioral treatment in TS including new approaches, for instance, attention training techniques (53).

### Research Organizations

There are several international scientific organizations dedicated to the coordination of research related to TS. An overview is given in Table 1.

**Table 1:** Examples of research initiatives in the field of Tourette syndrome.

	**Name *(Abbreviation)***	**Countries involved**	**Year of Foundation**	**Objectives/aims**	**Notes**
**Scientific organizations**					
1	Tourette Syndrome Association International Consortium for Genetics *(TSAICG)*	Canada, Germany, Netherlands, UK, USA	1986	Research on the genetic underpinnings of TS	
2	The European Society for the Study of Tourette Syndrome *(ESSTS)*	Pan-European	2000	Promoting research, educating professionals, patients & relatives, create public awareness	
3	The European Network for the Study of Gilles de la Tourette Syndrome	Pan-European	2010	Share knowledge, improve pan-European coordination of research, create public awareness	Inactive
4	TEC4Tic Research Unit	Germany, Hungary	2019	Investigation of the role of perception-action integration in TS	
5	Gilles de la Tourette Syndrome GWAs Replication Initiative *(GGRI)*	Austria, Canada, France, Germany, Greece, Hungary, Italy, Poland, USA		Collaboration and data sharing for genome-wide association studies	
6	Tic Disorders and Tourette Syndrome Study Group	Canada, France, Germany, Netherlands, Spain, UK, USA	2019	Increasing international collaborative research on TS, research on epidemiology and pathophysiology of TS	
7	Tourette Syndrome Genetics - The Southern and Eastern Europe Initiative *(TSGeneSEE)*	Albania, Greece, Hungary, Italy, Poland, Russia, Ukraine		Research on TS genomics, building a central repository of biomedical data	Inactive
**Multi-center studies**					
8	European Multicenter Tics in Children Studies *(EMTICS)*	Pan-European	2013	Examine the role of environmental factors on TS onset and course	
9	TS-Eurotrain	Pan-European	2012	Investigating the etiology and pathophysiology of TS	Inactive
10	Tourette International Collaborative Genetics Study *(TIC Genetics)*	Europe, South Korea, USA	2011	Sharing clinical data and biomaterials from patients and relatives, research on genetic architecture of tic disorders, building a central repository of biomedical data	

The European Society for The Study of Tourette Syndrome (ESSTS) is a pan-European society initially founded in Copenhagen, Denmark, in 2000 by Professor Mary Robertson. Its main aims include promoting research, educating professionals, patients, and their relatives, and creating awareness of TS. This is achieved, for example, by providing funds, organizing targeted events like training schools transferring knowledge to young doctors or researchers, or developing best-practice guidelines for the diagnosis and treatment of TS (21, 54–56). Meetings are held annually, and officers are elected periodically. Another important area of activity is developing alliances with patient support groups. The success of these efforts is evidenced by the fact that in 2012 “The First International Meeting of Tourette Syndrome Support Groups” took place. Particularly noteworthy was the cooperation between ESSTS and the European Cooperation in Science and Technology (COST), which in turn is a funding organization providing funds for scientists all over Europe. The European Network for the Study of Gilles de la Tourette syndrome, established in 2010 and active until 2014, can be regarded as one of the outcomes of this cooperation, aiming to share knowledge, to improve coordination of research at a European level, and to create public awareness of this disease (57).

In 2011, ESSTS was awarded a European grant amounting to €6 million with the objective to promote the investigations of immunological, infectious, and genetic processes in children and adolescents suffering from TS (https://cordis.europa.eu/article/id/92780-linking-tic-disorders-with-infection/de). In this context, in January 2013, a longitudinal observational multicenter study, the European Multicenter Tics in Children Studies (EMTICS) involving 17 clinical centers, were initiated (58). EMTICS was designed to examine the new appearance of tics within a group of children and adolescents with first-degree relatives suffering from tics, as well as the course of tics in previously diagnosed cases. Therefore, this study is composed of two different arms called “ONSET” and “COURSE” (58). The ONSET cohort encloses 260 children aged between 3 and 10 years, the COURSE cohort includes 715 children and adolescents between the ages of 3 and 16 years (58). The main focus of this study is the role of environmental factors including new infections caused by group A *Streptococcus*, related autoimmune processes, or group A *Streptococcus* carriage status on tic onset or course (58). Furthermore, the impact of other recent infections, psychosocial stress and pre- and perinatal adversities are being looked at (58). EMTICS is the largest observational study investigating new-onset tics within an at-risk population and the course of TS in already affected patients (58). The study was terminated in June 2018. The as-yet most relevant finding is that there is no evidence to indicate a relationship between new group A *Streptococcus* infections and the onset or course of tics (https://cordis.europa.eu/project/id/278367/reporting) (59). In addition to these important insights, through close cooperation of many different clinical centers all over Europe, new infrastructure and cooperation have developed (58).

With support from the European Commission, a research group of the ESSTS, the Marie Curie Training Network called TS-Eurotrain (Structuring EUROpean TRAINing capacities for neurodevelopmental disorders) was founded in 2012 with the objective to set up a database on genetic and environmental factors underlying TS (60).

Against the background of accumulating evidence suggesting that TS is predominantly a genetically determined disorder (61, 62), several national and international initiative consortia have been founded with the aim to unravel the genetic basis of TS.

As early as 1986, the Tourette Syndrome Association International Consortium for Genetics (TSAICG) was founded bringing together genetic researchers from the Netherlands and the USA to exchange knowledge and data (63). Initial projects focused on chromosomal aberrations or mutations in single genes (64, 65). After realizing that TS is not a monogenetic disease, the consortium was enlarged to include additional sites in the USA, Canada, Germany, the UK, and the Netherlands (63). Joint endeavors have led to the discovery of a number of rare variants of pathogenic relevance in individual families or small cohorts (66–68).

In this context, genome-wide association studies (GWAs), are of prime importance. Using this method, the whole genome is analyzed looking for intragroup variations in genomic DNA in the form of an altered single nucleotide. They are referred to as single nucleotide polymorphisms and are stored in databanks in single centers. It rapidly became apparent that sample sizes of single centers were far too small to obtain reliable results. Therefore, the different centers started to join forces combining their data in meta-analyses (69).

This progress has further fostered the development of large cooperative networks and open-access repositories (70). This resulted in the foundation of the Gilles de la Tourette Syndrome GWAs Replication Initiative (GGRI).

Likewise, the Tourette International Collaborative Genetics (TIC Genetics) Study funded by the American National Institute of Mental Health was launched in 2011. This ongoing project comprises more than 20 sites from the USA, Europe, and South Korea (70). By sharing biomedical data for GWAs, TIC Genetics and TSAICG closely cooperate (63). TIC Genetics follows two main approaches. First, genetic alterations shared by affected individuals within affected families are analyzed focusing on familial genetic variants. Second, trios, consisting of TS patients and their unaffected parents are investigated to identify *de novo* mutations using exome sequencing. Data collected in this way also allow to draw conclusions on the interaction between perinatal environmental factors and genetic alterations (70). Medical and biomedical data collected from ~2,000 people is stored in a shared repository domiciled within the National Institute of Mental Health Center for Collaborative Genomics Research on Mental Disorders, USA, and is accessible to a broader scientific community. This approach has been very fruitful, leading, for instance, to the discovery that *de novo* likely gene disrupting variants and copy number variations contribute to the genetic risk in TS (68, 71).

Another network focusing on genetic investigations in TS was established in Southern and Eastern Europe encompassing researchers from seven different countries (Greece, Hungary, Italy, Albania, Poland, Russia, and Ukraine), called Tourette Syndrome Genetics-The Southern and Eastern Europe Initiative (TSGeneSEE). Similar to TIC genetics, its objective was to build a central repository of biomedical data, predominantly based on trio whole exome sequencing in *de novo* TS patients and their parents enabling scientists to further investigate genetic variants associated with TS. It is not active anymore. Data are stored in a preexisting databank in Hungary (http://tsgenesee.mbg.duth.gr/index.html).

The Tic Disorders and Tourette Syndrome Study Group of the International Parkinson and Movement Disorder Society (https://www.movementdisorders.org/MDS/About/Committees--Other-Groups/Study-Groups/Tic-Disorders-and-Tourette-Syndrome-Study-Group.htm) aims at joining efforts to increase international collaborative research with regard to epidemiology and pathophysiology of tic disorders, enhancing the identification of biomarkers, investigating the efficacy and safety of novel treatment approaches, and accelerating the route toward personalized treatment plans by improving patient selection and increasing access to established treatments. More specifically, the group is currently working toward a consensus definition of tics and addresses perception and knowledge on tic disorders across the international plenum of movement disorders professionals with a particular view to clinical presentation, pathophysiology, assessment methods and tools, and treatment methods, including access to different types of treatment. Also, it aims at developing recommendations of instruments to capture comorbid conditions for clinical and research purposes and operationalizing clinically and scientifically relevant definitions, including, for instance, refractoriness to treatment. The study group includes adult and pediatric neurologists, child/adolescent and adult psychiatrists, neuropsychologists, neurosurgeons, as well as scientists involved in TS research, e.g., computational neuroscientists, behavioral scientists, and pharmacologists. The group aims also to include representatives of health professionals, in particular behavioral therapists, social workers, psychologists, and occupational therapists.

The TEC4Tic Research Unit (Cognitive Theory for Tourette syndrome—a novel perspective) (https://www.tec4tic.uni-luebeck.de) founded in 2019 and funded by the German Research Foundation (Deutsche Forschungsgemeinschaft, DFG, FOR 2698) comprised researchers from different fields, i.e., neurology, child/adolescent and adult psychiatry, (neuro-)pediatrics, cognitive and experimental psychology, neurophysiology, mathematics, and computational neuroscience based at the Universities of Lübeck, Dresden (Germany) and Budapest (Hungary). The Unit has been set up in the framework of the theory of event coding representing a cognitive theory for perception-action integration paying particular attention to their interdependency (72). The core hypothesis is that binding, or coupling, between perceptions and actions is particularly strong in TS (3, 43, 73, 74), because, clinically, there is a strong link between motor phenomena (tics) and perceptual abnormalities (premonitory urges preceding tics) (6). In addition to EEG, the Research Unit also applies functional and structural imaging, neuronavigated transcranial magnetic stimulation, electrical stimulation, and functional near-infrared spectroscopy addressing perception-action processing in different domains (visual and somatosensory), studying the neuropharmacology and developmental trajectories of perception-action processing, investigating effects of the social context on binding and also delineating the neural basis of coprophenomena.

## Summary

Given its complex phenomenology, etiology, and pathophysiology requiring expertise from different clinical disciplines including neurology, psychiatry, child and adolescent psychiatry, and adult psychiatry, as well as different research area, for instance, neuroanatomy, neurophysiology, neurogenetics, and cognitive psychology, both clinical care and research activities in TS need to be organized and structured in multidisciplinary, multiprofessional, and globally interconnected networks. A number of overarching umbrella organizations like the TSAICG and ESSTS coordinating international research and ensuring an exchange of information between groups already achieve these goals.

For many future research projects, particularly those requiring large amounts of data, e.g., genetic or brain imaging studies, successful realization, i.e., generation of valid and meaningful data, will crucially depend on international cooperation within structured and mutually beneficial networks. Against the background of the developments and achievements outlined in this review, this has now become a very realistic scenario, not least because of an overall friendly and supportive atmosphere and attitude in the field of TS research.

## Author Contributions

AK, MK, and AM: gathering information, writing of the first draft, and review and critique. AB: gathering information, AW, TB, CB, and VR: review and critique. All authors: contributed to the article and approved the submitted version.

## Conflict of Interest

The authors declare that the research was conducted in the absence of any commercial or financial relationships that could be construed as a potential conflict of interest. The Handling Editor declared a past co-authorship with one of the authors AM.

## Abbreviations

COST: Cooperation in Science and Technology
EMTICS: European Multicenter Tics in Children Studies
ESSTS: European Society for The Study of Tourette Syndrome
GGRI: Gilles de la Tourette Syndrome GWAs Replication Initiative
GWA: genome-wide association study
TAA: The Tourette Association of America
TIC Genetics: Tourette International Collaborative Genetics
TS: Tourette syndrome
TSAICG: Tourette Syndrome Association International Consortium for Genetics
TS-Eurotrain: Structuring EUROpean TRAINing capacities for neurodevelopmental disorders
TSGeneSEE: Tourette Syndrome Genetics-The Southern and Eastern Europe Initiative.
